# Lactate as Key Metabolite in Prostate Cancer Progression: What Are the Clinical Implications?

**DOI:** 10.3390/cancers15133473

**Published:** 2023-07-03

**Authors:** Paolo Chetta, Renuka Sriram, Giorgia Zadra

**Affiliations:** 1 Department of Pathology, Massachusetts General Hospital, Boston, MA 02114, USA; paolo.chetta.23@gmail.com; 2 Department of Radiology and Biomedical Imaging, University of California San Francisco, San Francisco, CA 94143, USA; renuka.sriram@ucsf.edu; 3 Institute of Molecular Genetics, National Research Council (IGM-CNR), 27100 Pavia, Italy

**Keywords:** prostate cancer, lactate, monocarboxylate transporters, tumor microenvironment, metabolic imaging, biomarkers

## Abstract

**Simple Summary:**

Advanced prostate cancer remains a clinical challenge, requiring novel therapeutic treatments and biomarkers for patient-risk stratification. From a metabolic perspective, primary prostate cancer shows unique features with respect to most solid tumors, being mainly characterized by an oxidative/lipogenic phenotype. However, increased aerobic glycolysis with enhanced lactate production (Warburg effect) is frequently observed in high-risk disease and during the onset of castration resistance. In this review, we summarize the key role of lactate in prostate cancer progression. We also highlight the clinical implication of lactate as a predictive/prognostic biomarker and the therapeutic potential of targeting lactate metabolism in prostate cancer.

**Abstract:**

Advanced prostate cancer represents the fifth leading cause of cancer death in men worldwide. Although androgen-receptor signaling is the major driver of the disease, evidence is accumulating that disease progression is supported by substantial metabolic changes. Alterations in de novo lipogenesis and fatty acid catabolism are consistently reported during prostate cancer development and progression in association with androgen-receptor signaling. Therefore, the term “lipogenic phenotype” is frequently used to describe the complex metabolic rewiring that occurs in prostate cancer. However, a new scenario has emerged in which lactate may play a major role. Alterations in oncogenes/tumor suppressors, androgen signaling, hypoxic conditions, and cells in the tumor microenvironment can promote aerobic glycolysis in prostate cancer cells and the release of lactate in the tumor microenvironment, favoring immune evasion and metastasis. As prostate cancer is composed of metabolically heterogenous cells, glycolytic prostate cancer cells or cancer-associated fibroblasts can also secrete lactate and create “symbiotic” interactions with oxidative prostate cancer cells via lactate shuttling to sustain disease progression. Here, we discuss the multifaceted role of lactate in prostate cancer progression, taking into account the influence of the systemic metabolic and gut microbiota. We call special attention to the clinical opportunities of imaging lactate accumulation for patient stratification and targeting lactate metabolism.

## 1. Introduction

Prostate cancer (PCa) is the second leading cause of cancer death in men in the US and the third in Europe [1,2]. Bone is the major site of secondary involvement, but lung and liver metastases are also common. Involvement of the liver is fostered by a favorable local microenvironment and is associated with an especially poor prognosis [3]. Androgen-receptor (AR) signaling is the driving force of PCa development and progression. While standard treatment for primary PCa is based on radical prostatectomy or radiotherapy, about 27–53% of cases relapse within a few years [4]. Recurrent tumors are usually treated with systemic androgen deprivation therapy (ADT) until they no longer respond. This condition is known as castration-resistant PCa (CRPC). CRPC cells are in fact capable of reactivating AR signaling despite negligible levels of circulating steroids and acquire AR-dependent specific metabolic vulnerabilities (discussed below). AR signaling reactivation occurs through several mechanisms including: (i) AR overexpression/amplification; (ii) AR gain-of-function point mutations and/or mutations that enhance AR activation by weak androgens, AR antagonists, and spurious ligands; (iii) expression of AR constitutive active splicing isoforms; (iv) alterations of AR co-regulators; and (v) activation of signal transduction pathways that enhance the AR response to low levels of androgens [5,6,7].

Patients with metastatic CRPC are commonly treated with second-generation AR antagonists (i.e., enzalutamide), intra-tumor androgen synthesis inhibitors (i.e., abiraterone), radium 223 chloride (approved in PCa patients with bone metastasis), taxane or platinum-based chemotherapy, poly(ADP-ribose)polymerase (PARP) inhibitors (approved in PCa patients with mutations in homologous recombination repair genes), and immune checkpoint inhibitors (approved in PCa patients with metastatic PCa harboring microsatellite instability or mismatch repair defects) [8]. While these therapeutic agents have prolonged life expectancy, none are curative [9]. Moreover, the outcomes from immunotherapy-based treatments have thus far been disappointing [10]. The situation is further aggravated by the emergence of very aggressive neuroendocrine variants that often occur after hormonal treatments. These variants display a loss of AR expression, gain of neuroendocrine or pro-neural markers (i.e., neuron-specific enolase and synaptophysin), and combined alterations in the tumor suppressors phosphatase and tensin homolog (PTEN), TP53, and RB transcriptional corepressor 1 (RB1) [11]. Neuroendocrine CRPC shows epithelial lineage plasticity and a poor response to available therapies [12]. The need to identify new actionable therapeutic targets for optimal patient management is compelling.

**Figure 1:**
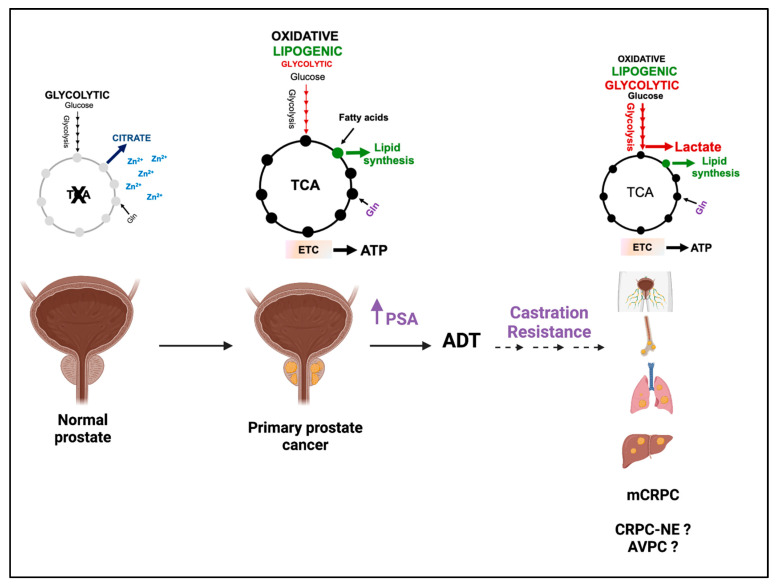
PCa acquires glycolytic features during the progression to metastatic castration-resistant PCa (mCRPC). In the normal prostate, high zinc levels inhibit the activity of the enzyme m-aconitase, inducing a truncated TCA cycle and substantial secretion of citrate in the spermatic fluid. In primary PCa, zinc levels are significantly reduced, such that PCa cells re-activate the TCA cycle and display an oxidative phenotype. Citrate is oxidized in TCA or exported to the cytosol for FA synthesis. As the disease progresses to mCRPC, both the TME and the AR signaling promote the expression of glycolytic genes and the acquisition of a glycolytic phenotype, resulting in an increased uptake of glucose and lactate accumulation. Lactate can also be produced from glutamine through glutaminolysis (highlighted in blue). In castration-resistant PCa with neuroendocrine features (CRPC-NE) and aggressive variant PCa (AVPC), the increase in aerobic glycolysis seems to be driven by AR-independent mechanisms. A clear understanding is, however, still missing (depicted with “?” symbol). ADT = androgen deprivation therapy. PSA = Prostate Specific Antigen. Violet arrow indicates increase in PSA. This figure was created with BioRender.com with license rights.

A substantial body of work supports the notion that AR signaling regulates the metabolic features of PCa during disease development and progression reviewed in [13,14]. Normal prostate cells take up exogenous fatty acids (FAs) and glucose and undergo a truncated tricarboxylic acid (TCA) cycle due to reduced m-aconitase activity. The enzyme m-aconitase is inhibited by high levels of zinc, which accumulates in the prostate through specific transporters that are highly expressed in the normal epithelium (i.e., ZIP1-4). In PCa, the expression of ZIP-1 is usually lost and the activity of m-aconitase is restored. Consequently, acetyl-CoA is oxidized to produce adenosine triphosphate (ATP) or exported into the cytosol to fuel de novo FA synthesis. Thus, during malignant transformation, energetically inefficient normal epithelial cells turn into energy/lipid-producing PCa cells (Figure 1) [15,16]. This explains the lack of utility of ^18^F-fluorodeoxyglucose (^18^F-FDG) positron emission tomography (PET) in detecting primary PCa [17]. In the last decade, the tight and mutual relationship between AR signaling and lipid metabolism has been confirmed. AR regulates the transcription of genes involved in lipid synthesis, transport, and catabolism at both early and advanced stages of the disease [18]. The AR signaling-mediated lipogenic phenotype is further exacerbated in advanced PCa and associated with the onset of castration resistance, as comprehensively discussed in previous reviews [13,14,19,20]. Analyzing metastatic CRPC, the lipogenic enzyme fatty acid synthase (FASN) emerged as one of the top genes co-expressed with AR-V7, highlighting the crucial role of de novo lipogenesis in mediating castration resistance [21]. Conversely, inhibitors of key lipogenic enzymes/transcription factors (i.e., FASN, sterol regulatory element-binding proteins [SREBP]) downregulate AR and AR-V7 [18,22]. In advanced PCa, many therapeutic strategies have targeted the lipogenic phenotype of PCa such as inhibitors of de novo FA synthesis (i.e., FASN, ATP citrate lyase, acetyl-CoA carboxylase), FA elongation (i.e., elongase of very-long fatty acid 5), FA desaturation (i.e., stearoyl-CoA desaturase 1), and FA oxidation (i.e., carnitine palmitoyltransferase 1) reviewed in [23]. The oral available FASN inhibitor TVB-2640 is currently tested in patients with CRPC in combination with enzalutamide (NCT05743621). However, as promising as these approaches may be, the advancement in analytic methods has uncovered PCa’s metabolic plasticity and dynamic adaptation to the available nutrients, hormonal levels, and microenvironmental conditions as the disease progresses. With the acquisition of castration resistance, androgen-independent activation of AR signaling or neuroendocrine transdifferentiation alters the canonical androgen-mediated regulation of cellular metabolism and forces cells to undergo drastic metabolic adaptation, including increased glucose uptake and enhanced glycolytic flux with pyruvate conversion to lactate, despite sufficient oxygen availability (Warburg effect or aerobic glycolysis) (Figure 1) [24,25,26]. Unsurprisingly, the increased expression of the glucose transporter 1 (GLUT1) has been reported in high-risk PCa associated with a poor prognosis and neuroendocrine features [27,28,29]. Other groups have reported the AR-dependent transcriptional upregulation of many components of the glycolytic pathway, including the enzyme lactate dehydrogenase A (LDHA) responsible for the conversion of pyruvate to lactate. Hyperpolarized (HP) magnetic resonance spectroscopic imaging (MRSI) confirmed an increased conversion of [1-^13^C] pyruvate to lactate in patient-derived xenografts (PDX) from AR-driven compared to AR-negative CRPC, suggesting AR-mediated modulation of aerobic glycolysis [30,31,32,33]. Thus, both AR-independent and -dependent mechanisms may contribute to increased aerobic glycolysis in advanced PCa.

In bone metastases, aerobic glycolysis seems to be promoted by adipocytes residing in the bone marrow metastatic niche [34], although a complete understanding of the role of the tumor microenvironment (TME) in dictating cancer-specific metabolic vulnerabilities in the metastatic niche is still missing.

In advanced and aggressive PCa, glutaminolysis can also contribute to lactate accumulation through the action of the cytosolic malic enzyme, which converts TCA cycle-derived malate to pyruvate. During this process, the concurrent generation of nicotinamide adenine dinucleotide phosphate (NADPH) equivalents can sustain FA synthesis. Glutaminolysis also acts as an anaplerotic source by providing carbon to the TCA cycle, while simultaneously contributing nitrogen for nucleotide synthesis. The relevance of glutamine metabolism in PCa progression and the potential clinical implications of targeting glutamine metabolism have already been comprehensively addressed elsewhere [35,36].

Here, we report the available evidence for increased aerobic glycolysis in PCa progression and the role of lactate (mostly generated by aerobic glycolysis) in TME modulation. We discuss the role of AR signaling and genetic alterations in promoting the “Warburg effect” during PCa progression We report the clinical usefulness of imaging lactate as a prognostic and/or predictive biomarker and current efforts in targeting lactate metabolism as a therapeutic strategy.

## 2. Lactate Concentration in PCa

The physiological concentration of lactate is about 1.5–3 mM in blood and healthy tissues. The lactate concentration in tumor tissues and tumor interstitial fluid is significantly higher, 10–20 mM: see Table 1 in [37]. In aggressive tumors, lactate levels can reach up to 40 mM [38]. The lactate concentration in biopsy tissues of PCa and normal prostate was measured using high-resolution magic angle spinning (HR-MAS) spectroscopy, resulting in values of 1.59 ± 0.61 and 0.61 ± 0.28 nmol/mg, respectively [39]. Additionally, a Gleason score-dependent increase in lactate concentration of 0.37 ± 0.06 mM in benign tissue; 0.65 ± 0.12 mM in low-grade PCa Gleason score ≤ 3 + 4); and 1.5 ± 0.35 mM in high-grade PCa (Gleason score ≥ 4 + 3) was observed and correlated with higher LDHA mRNA levels [40].

While the precise measurement of lactate intracellular/extracellular concentrations in the same PCa tissue section has not yet been performed, lactate efflux from benign and PCa living human biopsies was assessed using an optimized rotary tissue-culture method. Following 24 h of culture, PCa biopsies had a significantly higher lactate efflux rate (0.55 ± 0.14 nmol/min/mg) compared to benign biopsies (0.31 ± 0.04 nmol/min/mg), confirming an increased extracellular lactate efflux [41]. Future experiments of high-resolution spatial metabolomics (i.e., matrix-assisted laser desorption ionization–mass spectrometry imaging [MALDI-MSI]) and single cell metabolomics will be instrumental in measuring the intracellular and extracellular lactate concentrations in the same tissue section and better elucidating the role of lactate in the prostate TME.

## 3. Lactate Synthesis/Transport and PCa Clinical Outcomes

The synthesis of lactate is catalyzed by cytosolic enzymes called lactate dehydrogenases (LDHs). LDHs consist of LDHA (also known as LDH-M, which is abundant in the skeletal muscle) and LDHB (also known as LDH-H, which is abundant in the heart). A and B subunits co-assemble at varying ratios to form two homotetrameric isoforms (LDH1 or LDHB [B4] and LDH5 or LDHA [A4]), and three heterotetrameric isoforms (LDH2 [B3A1], LDH3 [B2A2], and LDH4 [B1A3]). All isoforms are able, with variable efficacy, to catalyze both the reduction of pyruvate to lactate and the oxidation of lactate to pyruvate [42]. LDHA has a low affinity for lactate and mainly catalyzes the conversion from pyruvate to lactate with the regeneration of nicotinamide adenine dinucleotide (NAD+), which provides the oxidative equivalents necessary to maintain the glycolytic flux and fuel glycolysis-associated pathways (i.e., pentose phosphate pathway) as reviewed in [43]. LDHB possesses a higher affinity for lactate and is involved in the conversion of lactate to pyruvate. A third type of LDH subunit, known as LDHC, is encoded by the *LDHC* gene and assembles solely into homotetramers to form the LDH6 isoform. LDH6 is mostly expressed in human testis and sperm and implicated in male fertility [44]. LDHA protein levels were found to be significantly increased in high-Gleason primary PCa and in metastases from a large cohort of clinical specimens (881 cases, 203 non-neoplastic, 176 high-grade prostatic intraepithelial neoplasia, 480 PCa adenocarcinomas, 10 metastatic PCa, and 12 benign prostates from cystoprostectomies) [45]. Furthermore, abnormal hyperphosphorylation or a high expression of LDHA and low expression of LDHB were found to be associated with a short overall survival and time to biochemical recurrence, defined as a rise in PSA level after prostatectomy or radiotherapy [46]. The authors described a fibroblast growth factor receptor (FGFR1)-dependent mechanism of LDHA overexpression whereby FGFR1 stabilizes LDHA protein through phosphorylation while repressing LDHB transcription. The latter is achieved by the inhibition of the demethylase TET1, resulting in LDHB promoter methylation and acquisition of highly glycolytic and aggressive features [46].

Several meta-analyses have been conducted to validate serum LDHA as a prognostic biomarker by examining the correlation of serum LDH with overall survival, cancer-specific survival, and progression-free survival in patients with metastatic PCa. A meta-analysis of 59 studies (19 from Europe, 21 from North America, and 19 from Asia) analyzed serum LDH levels in patients with metastatic CRPC and metastatic hormone-sensitive PCa [47]. The authors reported that high serum LDH was associated with a worse overall survival, regardless of whether all patients were pooled together or grouped separately into metastatic CRPC and metastatic hormone-sensitive PCa. Furthermore, LDH was associated with a worse overall survival in CRPC patients treated with either docetaxel or AR-signaling inhibitors. Overall, these observations support the notion that serum LDH, which can be easily and non-invasively measured during a routine diagnostic workup, may be considered as a prognostic tool to guide treatment decisions.

To avoid intracellular lactate accumulation, cancer cells export lactate in symport with protons into the TME through monocarboxylate transporters (MCTs). MCT-1, which has a higher affinity for lactate, is mainly involved in the uptake and import of lactate into the cell, whereas MCT-4 is mostly involved in lactate efflux. Immunohistochemical analyses of MCT-1 and MCT-4 in a well-annotated specimen collection including PCa/adjacent non-neoplastic tissues, prostatic intraepithelial neoplasia lesions, and normal prostatic tissues uncovered a significant increase in MCT-4 and decrease in MCT-1 in PCa cells compared to adjacent normal epithelium. MCT-4 expression was positively correlated with higher PSA levels, Gleason score, pT stage, perineural invasion, and biochemical recurrence [48].

While accumulating evidence supports lactate metabolism as an actionable metabolic adaptation and LDHA/MCTs as predictive and prognostic biomarkers, their clinical translation has only recently been actively investigated. We believe that the implementation of real-time, non-invasive metabolic imaging during diagnostic workup and therapy follow-up will be crucial in this context.

## 4. Regulation of Aerobic Glycolysis and Lactate Synthesis during PCa Progression

Aerobic glycolysis and lactate synthesis/transport are fine-tuned by AR signaling and alterations in oncogene and tumor suppressors that usually occur in CRPC. These metabolic adaptations support PCa progression, as discussed below.

### 4.1. Androgen-Receptor Signaling Promotes Aerobic Glycolysis and Lactate Synthesis during PCa Progression

Early evidence that AR signaling directly modulates the expression of glycolytic genes emerged from the work of Massie and coworkers in 2011 [32]. Using an interdisciplinary approach that integrates epi/genetic and metabolomics analyses, the authors found that AR directly upregulates the expression of key enzymes involved in glucose uptake and glycolysis (i.e., GLUT1, hexokinases 1 and 2, and 6-phosphofructo-2-kinase/fructose-2,6-bisphosphatase). The authors also showed that androgens stimulate glucose uptake and lactate production in normoxia through the activity of calcium/calmodulin-dependent kinase kinase 2 (CaMKK2), a newly discovered AR target gene. CaMKK2-dependent activation of the energy sensor AMP-regulated kinase led to the increased activity of the rate-limiting glycolytic enzyme phosphofructokinase 2, resulting in increased glucose uptake and lactate accumulation. Conversely, CaMKK2 knockdown or pharmacological inhibition with STO-609 was sufficient to block AR-stimulated glucose uptake and lactate production. This study uncovered CAMKK2 as an essential regulator of AR-induced aerobic glycolysis [32]. In a second landmark study, the same group undertook a comprehensive analysis of AR binding sites in PCa cell lines and clinical CRPC specimens and identified CRPC-specific AR binding sites. Gene ontology analyses identified enrichment of glucose homeostasis genes close to CRPC-specific AR binding sites, supporting the AR-mediated regulation of glycolytic genes in castration resistance [49]. Since AR-V7 regulates some canonical AR target genes, as well as a unique set of genes [50], the authors compared AR- and AR-V7-mediated regulation of metabolic pathways and showed that both AR and AR-V7 promote aerobic glycolysis and lactate accumulation in CRPC, suggesting that AR-V7 may have a critical role in driving the glycolytic shift in castration resistance. These findings highlighted a ligand-independent, AR-signaling-dependent stimulation of lactate production and pointed to aerobic glycolysis as an actionable metabolic vulnerability in AR-V7-positive metastatic CRPC [51].

In vivo confirmation of the AR- and AR-V7-dependent regulation of lactate synthesis in CRPC was independently obtained from data of hyperpolarized (HP) [1-^13^C]pyruvate, nuclear magnetic resonance (NMR) spectroscopy, and mass spectrometry (MS) performed in AR-dependent CRPC PDX models (AR/AR-V7-positive sublines MDA PCa 180 and 133-4 [52]) and AR-negative carcinomas with neuroendocrine features (sublines MDA PCa 155-2 and 144-13 [53]) [30]. An enhanced conversion of [1-^13^C]pyruvate to lactate and increased lactate levels were observed in AR-dependent CRPC PDX. Similarly, the [1-^13^C]pyruvate^-^to-lactate conversion rate and lactate levels were observed in the AR-dependent 133-4 PDX grown in either intact or castrated mice, suggesting that the increased lactate synthesis in CRPC is not dependent on androgen levels but most likely relies on AR signaling. These data also suggest that lineage plasticity in neuroendocrine CRPC might favor mitochondrial energy production and TCA-dependent anabolic processes, although others have reported otherwise (see above) [28,29].

**Figure 2:**
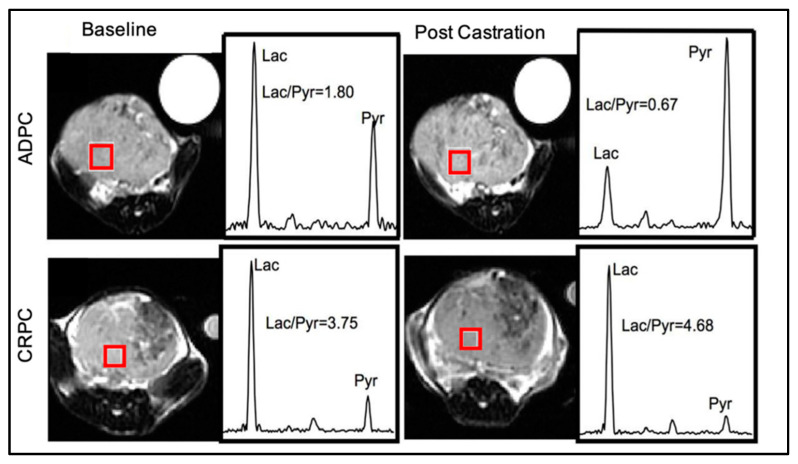
HP [1-^13^C]pyruvate MRSI uncovers increased aerobic glycolysis in CRPC. Representative T2-weighted proton images of androgen-dependent (ADPC, **top**) and castration-resistant (CRPC, **bottom**) TRAMP tumors at baseline (**left** column) and five days post-castration (**right** column). A T2-weighted image, a spin echo based pulse sequence in magnetic resonance imaging, provides contrast based on the transverse relaxation property of the water molecule which is dictated by the tissue properties and is used to differentiate the tumor from the surrounding normal tissue. The spectrum of the HP lactate (Lac) and pyruvate (Pyr) is shown on the right of each T2-weighted proton image. The Lac/Pyr ratio calculated from a voxel (red square) is reported. Image from Sun J et al. [54] with the author’s permission.

The transgenic adenocarcinoma of the mouse prostate (TRAMP), a well-established model of PCa progression, demonstrates increased aerobic glycolysis and lactate synthesis during the transition from androgen dependence to castration resistance [54]. In vivo U-^13^C glucose/^13^C glutamine flux analysis and HP [1-^13^C]pyruvate MRSI were performed together with ex vivo measurements of steady-state metabolites, enzymatic activities, and oxygen consumption/extracellular acidification rates. Castration-resistant TRAMP tumors (measured five days after surgical castration) showed increased levels of U-^13^C glucose-derived lactate, increased lactate/reduced citrate steady-state levels, and a higher LDHA activity compared to androgen-sensitive TRAMP tumors. These metabolic features were associated with increased HP [1-^13^C]pyruvate-to-lactate conversion (high HP Lac/Pyr ratio) (Figure 2), proving that HP [1-^13^C]pyruvate can noninvasively capture enhanced aerobic glycolysis in CRPC [54].

This study provided evidence for increased aerobic glycolysis (increased glycolytic flux and pyruvate reduction to lactate) in CRPC and proposed the calculated ratio of HP Lac/Pyr as a potential predictor of resistance to ADT. However, increased glutaminolysis (a potential glucose-independent source of lactate), anaplerosis into the TCA cycle, and glutathione synthesis were also observed in CRPC TRAMP tumors, suggesting that CRPC undergoes a complex metabolic rewiring that extends beyond aerobic glycolysis. Since TRAMP tumors display neuroendocrine features that can affect metabolic dependencies, studies in mouse models are being sought that more closely recapitulate human prostate adenocarcinoma [55].

Overall, HP MRSI studies have spotlighted aerobic glycolysis as a hallmark of CRPC but also highlighted the complex and still not fully understood crosstalk between AR signaling/aerobic glycolysis/lactate metabolism. Integrating spatial transcriptomics and metabolomics under ADT will be instrumental for a better definition of the AR-dependent modulation of glucose and lactate metabolism, considering tissue heterogeneity and clonal evolution.

### 4.2. Alterations in Oncogenes and Tumor Suppressors Modulate Aerobic Glycolysis and Lactate Synthesis in CRPC

Oncogenes and tumor suppressors dictate specific metabolic dependencies. Metastatic CRPC is predominantly characterized by *PTEN* loss, TP53 loss/mutations, c-MYC/8q24 amplification, mutations in DNA damage response genes (i.e., *BRCA1*, *BRCA2*, and *ATM*), and gain-of-function mutations in phosphoinositide-3 kinase (PI3K)/AKT-1 [56,57].

PTEN and TP53 co-deletions/mutations are associated with high levels of the glycolytic enzyme hexokinase 2 in PCa cell lines, xenografts, genetically engineered mouse models (GEMMs), and clinical samples. On the one hand, PTEN loss leads to the activation of the mTOR signaling pathway and 4EBP1 phosphorylation, resulting in the cap-dependent translation of hexokinase 2 [58]. On the other hand, hexokinase-2 expression is further increased by the reduced expression of miR-143, whose biogenesis is promoted by TP53 [59]. As discussed above, ^13^C MRSI data have confirmed increased lactate synthesis/efflux upon PTEN and TP53 inactivation, a feature that is further enhanced under castration [54]. Integrating metabolomics data from PCa cell lines and GEMMs has uncovered enhanced aerobic glycolysis and increased lactate levels in cells and mouse models that constitutively express AKT-1 compared to those overexpressing c-MYC. This was recapitulated in human tumors that express high levels of phosphorylated AKT-1 with respect to those with c-MYC overexpression [60]. While c-MYC is known to induce the expression of glycolytic genes and LDHA in several tumor types, neither an enrichment of glycolytic signatures nor increased lactate levels were observed in MYC-transformed ventral prostate compared to wild-type prostate, suggesting tumor/context-specific MYC-driven metabolic vulnerabilities [61]. The deficiency of the DNA damage response gene ATM increases LDHA expression and lactate levels to sustain CRPC cell growth. Conversely, LDHA genetic or pharmacological suppression with siRNA or the FX11 compound reduces lactate accumulation and promotes radical oxygen species (ROS)-induced cell death in ATM-deficient CRPC cells [62].

Altogether, these studies support aerobic glycolysis and lactate metabolism as actionable metabolic vulnerabilities in metastatic CRPC, especially in cases characterized by PTEN/p53 loss and defects in DNA damage response genes. Since patient selection and therapy follow-up can be achieved non-invasively with PET and HP ^13^C MRSI approaches, targeting aerobic glycolysis and lactate metabolism holds great potential for precision medicine.

## 5. PCa-Released Lactate Functions as a TME Modulator and Signaling Molecule

**Figure 3:**
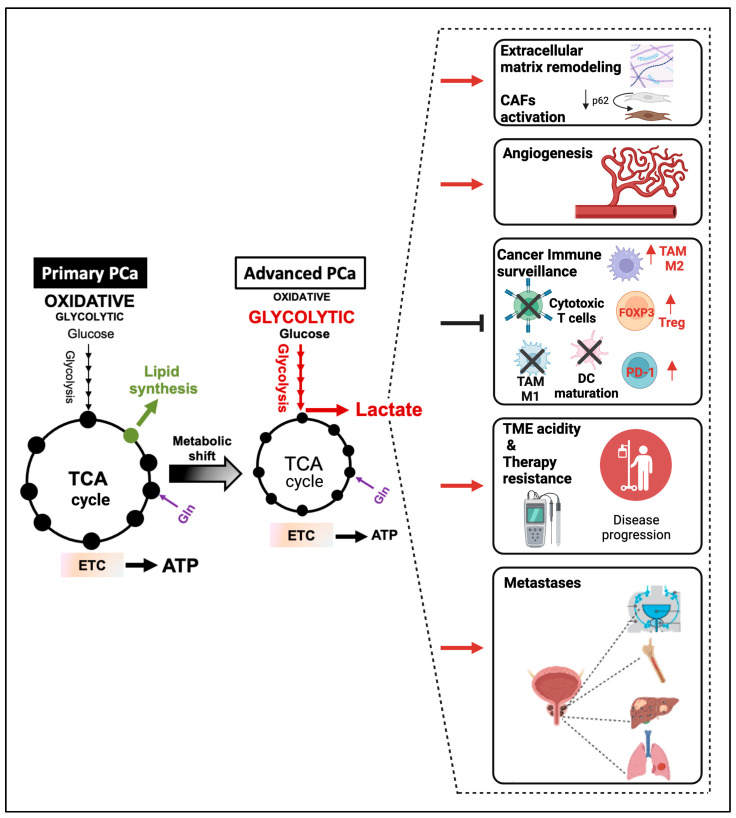
Lactate modulates the tumor microenvironment and promotes PCa progression. PCa undergoes a metabolic shift to adapt to the energetic requirements and nutrient availability as the disease progresses. Once produced, lactate is secreted into the TME where it promotes extracellular matrix (ECM) remodeling, neo-angiogenesis, immune evasion, TME acidity to favor cancer cell migration, invasion, metastasis formation, and therapy resistance. DC = dendritic cell, CAFs = cancer-associated fibroblasts, ETC = electron transport chain, TAM = tumor associated fibroblast, TCA = tricarboxylic acid, TME = tumor microenvironment, Treg = regulatory T cells, Vertical red arrow means increase. This figure was created with BioRender.com with license rights.

It is well established that lactate plays a key role in promoting cancer cell migration/invasion, angiogenesis, extracellular matrix (ECM) remodeling, immunological escape, metastasis formation, and resistance to therapy, as reviewed in [37,63,64,65,66,67] (Figure 3) This is achieved by lactate functioning as a metabolic fuel, signaling molecule, and metabolic/epigenetic regulator (see below). The lactate-induced aggressive phenotype is especially enhanced under hypoxic conditions, which usually occur during cancer progression. Hypoxia stabilizes the transcription factor hypoxia-inducible factor 1 alpha (HIF-1α) and promotes the expression of glycolytic genes, including LDHA and MCT-4 as reviewed in [68], which favor lactate synthesis and secretion. Conversely, lactate promotes HIF-1α stabilization (see below). Here, we summarize the most recent evidence of lactate as a TME modulator and signaling molecule to promote PCa progression.

### 5.1. PCa-Derived Lactate Induces an Immunosuppressive and Metastasis-Supportive TME

In the last decade, accumulating evidence has uncovered lactate as a key metabolite in modulating both innate and adaptative immune responses. This occurs through several mechanisms, including the polarization of macrophages towards a pro-tumorigenic M2-like phenotype, abrogation of natural killer (NK) cell activity, inhibition of dendritic cell maturation and antigen-presenting function, suppression of CD8+ cytotoxic T-cell activity and induction of the anergy state, and enhanced proliferation, recruitment, and activity of regulatory T cells (Treg). Following activation, CD8+ cytotoxic T cells rely on aerobic glycolysis and lactate secretion for their proliferation and functional activity. A high concentration of lactate in TME creates a gradient that prevents lactate secretion from CD8+ cytotoxic T cells. As a result, intracellular lactate buildup inhibits the activity of glycolytic and other enzymes, impairing the proliferation of CD8+ cytotoxic T cells and their capability to secrete interferon γ and other anti-tumor cytokines [69]. In contrast, a high concentration of lactate in TME serves as fuel for oxidative Treg cells. The Treg transcription factor forkhead box P3 (foxp3) suppresses c-MYC signaling and shifts Tregs towards an oxidative metabolism, allowing them to cope with a low-glucose and high-lactate microenvironment and escape anti-tumor immune surveillance [70]. In tumor-associated macrophages (TAMs), lactate induces HIF-1α stabilization and the HIF-1α-dependent/independent expression of M2-polarizing genes (i.e., Arg and VEGF) that promote an M2-like phenotype [71]. Finally, lactate has been implicated in inhibiting the anticancer activity of NK cells. Lactate can directly inhibit NK cytolytic function and the expression of perforin, granzymes, and NKp46, or indirectly blunt NK cell function through the recruitment of monocyte-derived dendritic cells [72] (Figure 3).

While the role of lactate as an immune modulator is well-established and the underpinning mechanisms have been comprehensively discussed in outstanding reviews [37,63,64,65,66,67], little is still known about the role of lactate in rewiring prostate immune TME. This is especially relevant since PCa is considered an immunologically “cold” cancer characterized by: (i) a low mutational burden and immunogenicity; (ii) immunosuppressive TME with a paucity of pre-existing T cells and a relative enrichment of innate immunosuppressive myeloid cells such as TAMs and myeloid-derived suppressor cells; and (iii) a limited response to immunotherapy. Thus, strategies to “turn up the heat” on the cold immune TME of metastatic PCa are being intensively sought. Using the PTEN/p53-deficient GEMM, a metastatic model that recapitulates the genomic alterations and the “cold” TME observed in human metastatic CRPC, Chaudagar and coworkers recently uncovered an immunosuppressive role for lactate through TAM epigenetic remodeling [73]. PCa-cell-derived lactate induces histone-3 acetylation on lysine 18 (H3K18lac) within TAMs, resulting in the suppression of their anti-cancer phagocytic activity [73]. Conversely, inhibiting PTEN/p53-deficient PCa-cell-derived lactate efflux using the PI3K inhibitor copanlisib reduces histone acylation and promotes TAM activation/phagocytosis. Consistently, single-cell RNA sequencing in patient biopsies of metastatic CRPC has revealed a direct correlation between tumor glycolytic activity and the suppression of TAM phagocytosis, confirming that enhanced tumor glycolysis blunts the TAM-mediated innate response within the metastatic prostate TME. The recruitment of PD1-expressing TAMs thwarts ADT/PI3Ki-mediated tumor control. Anti-PD1 antibody addition led to a TAM-dependent, approximately three-fold increase in the anti-cancer response, suggesting that immunometabolic strategies that reverse lactate and PD1-mediated TAM immunosuppression, in combination with ADT, warrant further investigation in PTEN-deficient metastatic CRPC patients [73].

Lactate secretion occurs in symport with protons, which reduces the TME pH. TME acidity is implicated in PCa progression and immune evasion by promoting a macrophage pro-tumorigenic phenotype [74].

PCa-derived lactate is also taken up by cancer-associated fibroblasts (CAFs) where it downregulates the transcriptional levels of p62 (a tumor suppressor in stromal cells) and induces stromal activation. Specifically, lactate is converted to pyruvate in CAFs, resulting in a decreased NAD+/NADH ratio and impaired PARP-1 activity. In turn, PARP-1 inhibition blocks the poly(ADP-ribosyl)ation of the AP-1 transcription factors c-FOS and c-JUN resulting in p62 downregulation, enhanced CAF activity, and PCa aggressiveness [75]. Since the PARP-1 inhibitors used in the clinic, such as olaparib, mimic the effect of lactate in downregulating p62 and promoting CAF activity, these data suggest that the efficacy of olaparib might be dampened by the activation of CAFs [75]. Future studies that explore the benefit of combining Olaparib with anti-stromal targeted therapies are being sought.

Cancer-cell-derived lactate can also contribute to ECM remodeling in TME. In certain cancers, lactate stimulates fibroblast expression of hyaluronan and its receptor CD44, resulting in an increased ECM stiffness that favors cancer cell migration, invasion, and metastasis formation [76]. Future studies are needed to confirm that this also occurs in CRPC.

Finally, cancer-cell-produced lactate also contributes to a metastasis-permissive milieu by promoting angiogenesis via both HIF-dependent and HIF-independent mechanisms [77,78]. HIF-independent mechanisms include the activation of a reactive oxygen species (ROS)-dependent NF-κB/IL-8 pathway [77] and the direct binding of the N-MYC downstream-regulated protein (NDRG3). Lactate binding to NDRG3 prevents protein degradation by the prolyl hydroxylase 2 (PHD2)/Von Hippel–Lindau tumor suppressor and promotes the activation of c-Raf-ERK signaling [79]. HIF-dependent mechanisms include the inactivation of PHD2 due to lactate competition with the PHD2 activator alpha-ketoglutarate (α-KG), preventing HIF-1α ubiquitination and degradation. Stabilized HIF-1α enhances the transcription of neoangiogenic factors, including VEGF, and promotes angiogenesis irrespective of the oxygen supply [78]. Recently, a new mechanism to promote angiogenesis through HIF-1α lactylation has been described in PCa. Following lysine lactylation, HIF-1α translocates to the nucleus to form a complex with HIF-1β and promotes the transcription of the hyaluronic acid binding protein KIAA1199. In turn, KIAA1199 favors VEGF-A synthesis/secretion, erythropoietin-producing hepatocellular A2 phosphorylation, and vascular endothelial-cadherin expression, resulting in increased angiogenesis and vascular mimicry [80].

While this investigation is still in its infancy, it is becoming clear that PCa-cell-generated lactate hijacks the TME to generate an environment permissive of metastatic spread and immune evasion.

### 5.2. Lactate as a Signaling Molecule and Modulator of Protein Activity

Aside from its metabolic role and immune modulatory function, lactate can also promote cancer progression by acting as a signaling molecule and regulator of enzyme and protein activity. Lactate binds to GRP81 (also known as hydroxycarboxylic acid receptor 1), a member of a subfamily of G protein-coupled receptors, which is upregulated in many cancers [81]. Cancer-cell-generated lactate activates GPR81 in both an autocrine and paracrine manner. Lactate binding to GPR81 in an autocrine manner promotes cancer cell migration/invasion, DNA repair, immune evasion, and chemoresistance in several cancer types. The main underpinning mechanisms are: (i) increased expression of the DNA repair proteins BRCA1, nibrin, and DNA-PKcs; (ii) increased expression of PD-L1; and (iii) increased expression and activity of the ABCB1 drug-exporting transporter [82,83]. Lactate binding to GPR81 in a paracrine manner promotes TAM polarization towards an M2-like phenotype, inhibits the expression of major histocompatibility complex (MHC) II on the cell surface of antigen-presenting dendritic cells, boosts angiogenesis in GPR81-expressing endothelial cells, and favors the release of hormones and pro-tumorigenic cytokines from TME-associated adipocytes as reviewed in [65]. In PCa, the role of lactate binding to GRP81 has not yet been investigated. Future studies are warranted in this area.

Lactate can also directly modulate the activity of targeted enzymes and proteins, including PHD2, histone deacetylases, and NDRG3 (discussed above).

In oxidative cancer cells, lactate taken up from the TME through MCT-1 competes with the PHD2 activator 2-oxoglutarate and inhibits the activity of PHD2. The suppression of PHD2 activity prevents HIF-1α hydroxylation and its proteasomal degradation in normoxic conditions. As a result, HIF-1α protein expression is stabilized and promotes tumor growth and angiogenesis [84].

Lactate inhibition of NAD+-independent histone deacetylases results in the deregulation of gene expression, especially the upregulation of genes associated with histone deacetylase proteins [85]. Thus, lactate has an important role as a transcriptional regulator, linking the metabolic state of a cancer cell to gene transcription. Not much is known regarding the influence of lactate on the PCa epigenome. Future studies are needed to better elucidate this aspect.

## 6. Metabolic Heterogeneity Supports “Symbiotic” Interactions through Lactate Shuttling to Sustain PCa Progression

**Figure 4:**
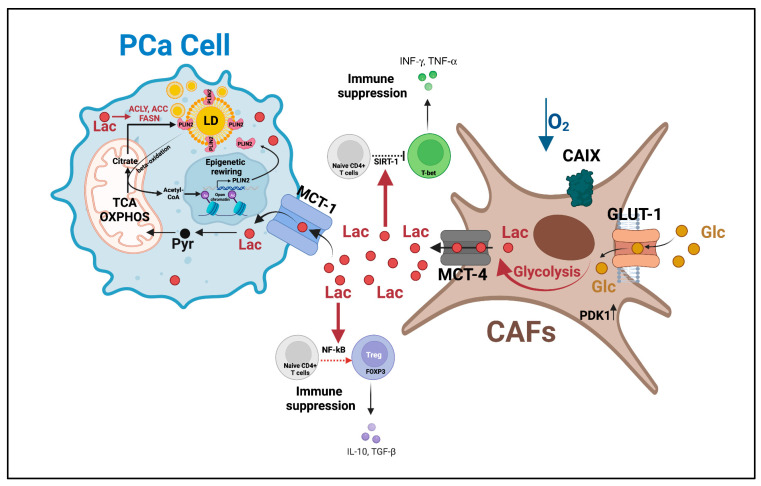
Lactate shuttle between CAFs and PCa cells promotes castration resistance and disease progression. Lactate shuttling between CAFs and PCa cells: (i) promotes mitochondrial activity to fulfill PCa cells’ enhanced energetic needs, (ii) inhibits immune surveillance, and (iii) induces epigenetic-regulated lipid metabolism rewiring. This multifaceted role of the lactate shuttle sustains PCa survival in harsh environments (i.e., hypoxia) and the acquisition of PCa invasive features. AC = acetylation, ACC = acetyl-CoA carboxylase, ACLY = ATP citrate lyase, CAFs = cancer-associated fibroblasts, CAIX = hypoxia-regulated carbonic anhydrase IX, FASN = fatty acid synthase, GLUT-1 = glucose transporter 1, Lac = lactate, LD = lipid droplets, MCT-1 = monocarboxylate transporter 1, OXPHOS = oxidative phosphorylation, PCa = prostate cancer, Pyr = pyruvate, PLIN2 = perilipin 2, SIRT-1 = sirtuin 1, TCA = tricarboxylic acid. This figure was created with BioRender.com with license rights.

While CRPC cells enhance aerobic glycolysis and secrete lactate to promote metastatic dissemination and immunosuppressive TME (as discussed above), both PCa and TME cells also display metabolic heterogeneity as the tumor grows at a fast rate outpacing the rate of blood vessel formation. In these poorly vascularized/hypoxic areas with a reduced nutrient supply, glycolytic cancer cells and CAFs convert the available glucose to lactate. Lactate is then secreted into the TME through MCT-4 and diffuses to more oxygenated peripheral areas. There, oxidative PCa cells take up lactate via MCT-1 and use it as fuel. Lactate is then converted back to pyruvate by LDHB and metabolized through the TCA cycle and oxidative phosphorylation to fulfill energetic needs. The lactate shuttling between glycolytic and oxidative PCa cells and, most frequently, between glycolytic CAFs and oxidative PCa cells represents an adaptation to nutrient- and oxygen-deprived environments to sustain cancer progression, as discussed in [86]. It has been proposed that an oxidative cancer cell can “instruct” adjacent CAFs to boost aerobic glycolysis [87]. The mechanisms underpinning this phenomenon, known as the “reverse Warburg effect”, are still under investigation. In vitro genetic or pharmacological inhibition of MCT-1 in PCa cells reduces PCa survival in glucose-restricted conditions, confirming the relevance of lactate shuttling for PCa progression [88,89]. Consistently, immunohistochemical analyses of human PCa tissues have shown an increased expression of MCT-4 and MCT-1 in CAFs and PCa cells, respectively. This molecular phenotype was associated with the disease stage and biochemical recurrence after surgery [90]. Three recent studies have provided additional clues for the role of lactate shuttling between CAFs and PCa cells in sustaining PCa progression [91,92,93]. In the first study, the authors reported that lactate uptake and conversion to pyruvate increases the NAD^+^/NADH ratio in PCa cells and promotes sirtuin-1-dependent activation of the proliferator-activated receptor-gamma coactivator 1 alpha, resulting in enhancement of mitochondrial mass/activity, altered expression of mitochondrial complexes, and deregulation of TCA cycle. The latter causes accumulation of the oncometabolites succinate and fumarate, promoting HIF-1α stabilization and development of invasive features in PCa cells. Enhanced mitochondrial activity and alteration in mitochondrial complexes further promote PCa aggressiveness through the generation of ROS and the activation of ROS-dependent signatures (i.e., Src signaling). Finally, CAFs can promote an invasive phenotype by directly supplying PCa cells with functional mitochondria through cellular bridges to boost their respiratory capacity and oxidative metabolism (Figure 4) [91]. In the second study, it was demonstrated that glycolytic CAF-released lactate is not only a fuel source but also exerts an immunomodulatory effect. Specifically, CAF-derived lactate reduces the percentage of the antitumoral Th1 CD4+ T-helper cell subset and increases that of Treg cells. The reduction in Th1 cells is achieved by the lactate-dependent activation of sirtuin 1 and subsequent deacetylation/degradation of the T-bet transcription factor. The increase in Tregs is mediated by the lactate-based NF-kB activation and induction of Foxp 3 expression, which promotes CD4+ naive T-cell polarization towards Treg. In turn, the immunosuppressive TME promotes EMT and invasiveness via activation of the microRNA-21/toll-like receptor 8/nuclear factor kappa B axis (Figure 4) [92]. In the third study, the authors unveiled a mechanism by which CAF-derived lactate increases the expression of genes involved in de novo FA synthesis (i.e., ATP citrate lyase, acetyl-CoA carboxylase, FASN), resulting in enhanced FA accumulation in lipid droplets. In turn, droplet lypolysis provides acetyl moieties for histone acetylation, promoting the transcription of the droplet component perilipin 2 (Figure 4). In human tissues, perilipin 2 is highly expressed in high-Gleason PCa and in CRPC. Conversely, the inhibition of the CAF-induced metabolic–epigenetic regulatory loop using the bromodomain and extra-terminal domain inhibitor I-BET762 reduces PCa growth and metastasis formation [93].

Finally, new data have uncovered CAF-derived lactate shuttling as a mediator of ECM remodeling. According to this recent study, CAF-derived lactate is taken up by PCa cells and oxidized to α-KG, which is used as a co-factor for the activity of the enzyme collagen prolyl-4-hydroxylase, resulting in increased collagen hydroxylation and deposition. Increased collagen deposition is involved in the activation of the discoidin-domain collagen receptor 1 and its downstream signaling via STAT3, which significantly enhances PCa cells’ invasive properties. CAF-derived lactate has also been shown to also boost the formation of anchorage-independent tumor clusters and transendothelial migration in vitro and in vivo, suggesting a potential role of collagen in circulating tumor cells and the metastatic dissemination of lactate-reprogrammed PCa cells [94].

Altogether, these findings have uncovered lactate shuttling as a metabolic fuel, immune/epigenetic modulator, and ECM remodeler to sustain PCa progression. Disrupting PCa–CAFs interaction holds potential as a therapeutic approach.

## 7. Systemic Metabolism and Gut Microbiota Affect Lactate Levels and PCa Progression

**Figure 5:**
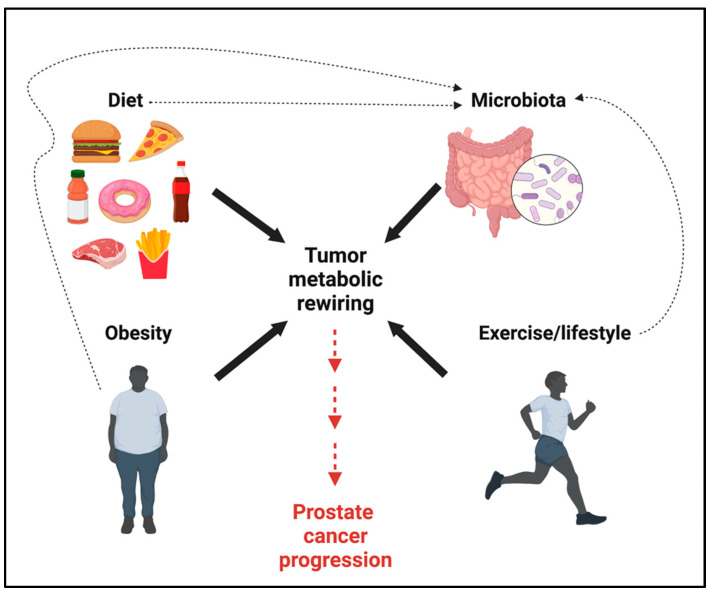
Diet, obesity, and lifestyle promote tumor metabolic rewiring. Diet, obesity, and lifestyle affect the gut microbiota and circulating/intra-tumor metabolite levels, promoting PCa progression. This figure was created with BioRender.com with license rights.

Diet and metabolic diseases, including obesity, affect the TME and disease progression (Figure 5) [95,96]. However, whether alterations in systemic metabolism promote the accumulation of specific metabolites in PCa and TME to foster PCa aggressiveness remains to be fully clarified. Recently, it has been shown that obesity increases the levels of lactate in MYC-driven PCa, promoting angiogenesis, the increased infiltration of Treg and M2-like TAMs, and PCa progression [97]. Both obesity and the increased consumption of sugar- and saturated fat-rich diets are associated with more aggressive and lethal PCa [23]. Thus, a better understanding of the effects of diet and obesity on cancer metabolome and TME modulation is of paramount importance to design approaches of precision nutrition to halt PCa progression. The inclusion of dietary interventions alongside standard oncological treatments is gaining more and more attention, as clinical trials have started to explore their benefits based on positive preclinical data. Several calorie-restricted diets, such as cyclic fasting-mimicking diet, have provided benefits in several tumor types [98,99]. In PCa patients, this diet can improve the anti-tumor immune response [100].

Metabolic diseases (i.e., obesity), diet (i.e., high fat diet), and drug exposure (i.e., antibiotics or ADT) also induce gut microbiota dysbiosis, which is associated with the promotion of PCa progression (Figure 5) [101,102,103].

The gut microbiota generates metabolites that circulate within the human host and affect cancer risk. For example, increased levels of the serum gut-microbiota-derived metabolites choline, trimethylamine N-oxide, phenylacetylglutamine have been associated with a diagnosis of aggressive PCa and increased risk of lethal PCa [104]. Furthermore, gut microbiota species that convert androgen precursors into active testosterone have been observed in CRPC patients and associated with resistance to ADT [102]. The concentration of lactate in the gut microbiota is kept in check by the balanced activity of lactate-producing bacteria (i.e., Lactobacilli, Streptococci, and Bifidobacteria) and lactate-metabolizing bacteria (i.e., Coprococcus catus) that prevent lactate accumulation in the colon, pH reduction, and dysbiosis. Lactate is metabolized to short-chain FAs (SCFAs), acetate, butyrate, and propionate by several gut obligately anaerobic bacteria of the firmicutes phylum, including the Lachnospiraceae family as reviewed in [105]. Lactate-metabolizing and SCFA-producing gut bacteria (i.e., Lachnospira, a genus of the Lachnospiraceae family) were found to be significantly increased in Japanese men with high-Gleason PCa. Additionally, starch and sucrose metabolism was found to be elevated in the gut microbiota of high-risk PCa patients, in line with a previous study conducted in the US [106,107]. An obesogenic high-fat diet was shown to enhance gut bacterial production of SCFAs and support PCa growth through an SCFA-mediated increase in systemic and local levels of insulin-like growth factor 1 [108]. Thus, it is likely that lactate-metabolizing bacteria may be involved in PCa progression by increasing SCFA levels.

However, confirmatory studies using multiracial populations with different lifestyles are strongly needed. Improvements in the integration of shotgun metagenome and metabolic analyses will be instrumental in clarifying the role of lactate-metabolizing gut bacteria in PCa progression.

In addition to the gut microbiome, the prostate and bladder microbiome, which can be easily assessed in the urine and prostatic fluid, can also affect PCa development and progression [109,110,111,112]. The specific role of prostate and bladder lactate-metabolizing bacteria in PCa progression has not yet been investigated. However, bacteria capable of metabolizing lactate (i.e., Lachnospiraceae) were found to be higher in the prostatic secretions of PCa patients and may help to uncover new underpinnings of PCa progression [113].

## 8. Imaging Glucose Uptake and Pyruvate-to-Lactate Conversion as Diagnostic, Prognostic, and Predictive Biomarkers

Given the evidence for a metabolic switch during PCa progression, extensive efforts have been dedicated to exploiting glucose metabolism rewiring and lactate levels for diagnostic and therapeutic purposes. These efforts have focused on repurposing ^18^F-FDG PET in metastatic CRPC and on developing HP ^13^C pyruvate MRSI to monitor pyruvate-to-lactate conversion in real time. The use of HP MRSI has significantly expanded our knowledge on lactate as a potential diagnostic, prognostic, and predictive biomarker.

### 8.1. Preclinical/Ex-Vivo Studies

^18^F-FDG PET measures neither the glycolytic flux nor the lactate synthesis per se. However, it can assess the uptake and accumulation of the non-metabolizable glucose analogue ^18^F-FDG, which often correlates with glycolytic flux and lactate synthesis in cancer. ^18^F-FDG PET shows a limited usefulness in the detection and staging of primary PCa, whose oxidative/lipogenic phenotype is better captured using precursor radionucleotides such ^11^C/^18^F-choline and ^11^C-acetate [17,114]. During castration resistance, PCa undergoes the glycolytic switch and enhances glucose uptake, which can be captured with ^18^F-FDG PET. On this basis, ^18^F-FDG PET can be exploited to monitor disease progression and predict the response to ADT before clinical effects are evident. Oyama et al. have indeed shown that ^18^F-FDG outperforms ^11^C-acetate in monitoring ADT efficacy in CWR22 xenograft tumors from mice that were exposed to ADT (diethylstilbestrol) or a vehicle for a week. While ^11^C-acetate uptake did not show a significant difference after ADT treatment, ^18^F-FDG uptake was already markedly reduced, suggesting that ^18^F-FDG PET may predict the ADT response [17]. However, results were not recapitulated in LAPC-4 (androgen-dependent) or 22Rv1 (androgen-independent) xenograft tumors from mice subjected to surgical castration for six days [115]. While it cannot be ruled out that the selected time point was too early to catch a reduction in the ^18^F-FDG uptake, the inconsistent preclinical data highlight the need to perform ad hoc clinical studies to truly establish the usefulness of ^18^F-FDG PET to predict and monitor the response to ADT, as discussed below.

More recently, the use of HP [1-^13^C] pyruvate MRSI has enabled the direct and non-invasive investigation of lactate as an early prognostic and predictive biomarker in preclinical/ex vivo studies. HP [1-^13^C] magnetic resonance (MR) is a powerful metabolic imaging method that uses a dynamic nuclear polarization technique to provide signal enhancements of over 10,000-fold for ^13^C-enriched, endogenous, non-radioactive compounds suitable for clinical translation for the real-time monitoring of enzyme kinetics. Contrary to ^18^F-FDG, which assesses only glucose uptake and phosphorylation, HP ^13^C MRSI using [1-^13^C]pyruvate detects the rate of conversion for [1-^13^C]pyruvate to [1-^13^C]lactate (also defined as k_PL_). The earliest application of this technology in an animal model of PCa, namely the TRAMP, demonstrated an increased pyruvate-to-lactate conversion in aggressive PCa [116]. This was also confirmed by using dual-agent ^13^C-pyruvate and ^13^C-urea MRSI and multi-parametric ^1^H magnetic resonance imaging (MRI) to investigate changes in both tumor perfusion and lactate metabolism in the TRAMP GEMM [117]. An elevated k_PL_ and a higher LDHA expression/activity were observed in high-versus low-grade TRAMP tumors compared with normal prostates. This co-occurred with the presence of a hypoxic TME in high-grade tumors, as measured by a decreased HP ^13^C urea perfusion and an increased expression of PIM-1, a protein that stabilizes HIF-1α and promotes LDHA and MCT-4 expression. Conversely, an LDHA-inducible knock-out significantly inhibited TRAMP tumor growth and the formation of lymph node and visceral metastasis, highlighting the need for LDHA activity and lactate synthesis in TRAMP tumor progression. These data were recapitulated in patient-derived tissue slices from prostatectomy specimens kept in an NMR-compatible three-dimensional tissue culture bioreactor, an ex vivo model that closely recapitulates the pathologic and biologic heterogeneity of human PCa. The authors confirmed that higher-Gleason PCa cases display increased k_PL_, LDHA, and MCT-4 expression/activity [40], suggesting that HP ^13^C MRI could be used to distinguish low-risk (Gleason score ≤ 3 + 4) from high-risk (Gleason score ≥ 4 + 3) PCa in the clinical setting, as discussed below. In addition, HP ^13^C MRI has been useful in detecting the response to different types of drugs that reduce aerobic glycolysis, independently of their specific target. These include inhibitors of PI3K, AKT, LDHA, platelet-derived growth factor imatinib, and ADT or radiation [54,118,119,120,121,122].

Altogether, these successful preclinical results provided the rationale for further clinical investigation, as discussed below.

### 8.2. Clinical Studies

#### 8.2.1. ^18^F-FDG PET

**Figure 6:**
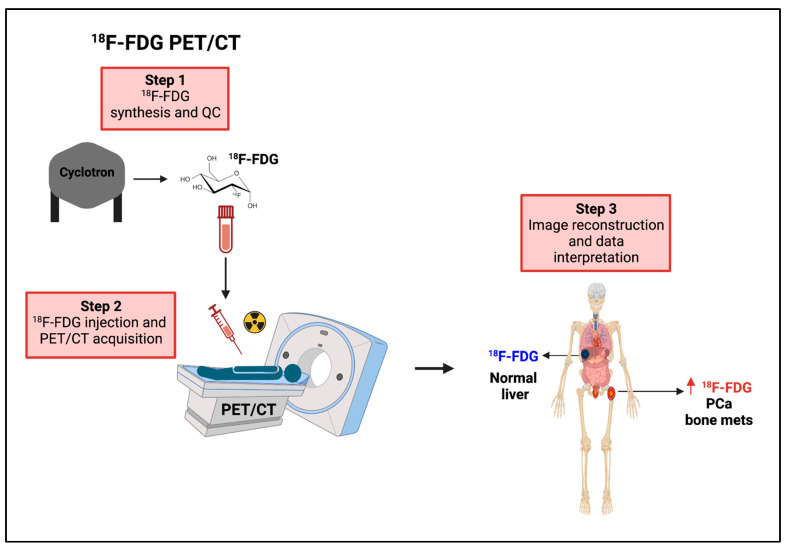
Non-invasive imaging of glucose uptake using ^18^F-FDG PET. Workflow of ^18^F-FDG PET in the clinical setting. ^18^F-FDG PET can provide useful information in the diagnostic workup and therapy decision making. CT = computerized tomography, ^18^F-FDG = [^1^⁸F]fluorodeoxyglucose, mets = metastases, PET = positron emission tomography, PCa = prostate cancer. Vertical red arrow means increase, This figure was created with BioRender.com with license rights.

In line with preclinical findings, ^18^F-FDG PET has regained attention as a non-invasive tool for assessing PCa progression in the clinical setting (Figure 6). Several studies have indeed reported the usefulness of ^18^F-FDG in detecting both recurrent PCa and metastatic CRPC reviewed in [123]. ^18^F-FDG-PET is especially useful in the detection of about 20% of high-Gleason score CRPCs that are negative under ^68^Ga-prostate-specific membrane antigen (PSMA) PET [124]. Although ^68^Ga-PSMA PET remains the imaging modality with the highest detection efficiency (sensitivity and specificity) in both localized and recurrent PCa [125,126,127], patients with high-Gleason CRPC and an elevated PSA may greatly benefit from additional ^18^F-FDG PET/computerized tomography (CT) [125]. Consistent with AR-mediated regulation of glucose metabolism (see above), ^18^F-FDG PET is a useful tool to monitor ADT efficacy in PCa patients. FDG accumulation was shown to be significantly reduced in PCa patients treated with ADT for 1–5 months, in line with a reduced serum PSA level and prostate size measured on CT [128]. In terms of predictive power, ^18^F-FDG uptake in both high-Gleason primary PCa and metastatic castration-sensitive PCa represents a useful predictor of biochemical recurrence. Indeed, preoperative intraprostatic FDG uptake was shown to predict castration resistance following radical prostatectomy in PCa patients with a Gleason score ≥ 8 at biopsy [129]. Similarly, the sum of the standardized ^18^F-FDG uptake value and the number of positive lesions was reported as an independent predictor of biochemical recurrence in patients with metastatic castration-sensitive PCa [130]. More recently, ^18^F-FDG PET imaging was conducted in a cohort of patients with metastatic CRPC, including a subgroup that received FDG-PET/CT before chemotherapy (i.e., docetaxel or cabazitaxel) or treatment with androgen-signaling inhibitors (i.e., abiraterone or enzalutamide). Both the total metabolic tumor volume and the total lesion glycolysis predicted a shorter overall survival. Furthermore, a lower total lesion glycolysis was associated with higher success rates for AR-targeted agents but had no impact on chemotherapy efficacy, suggesting that ^18^F-FDG PET not only holds a prognostic value in metastatic CRPC but is also useful in predicting response to treatment and guiding treatment decision making [131].

Altogether, these studies support: (i) the diagnostic use of preoperative FDG-PET/CT to distinguish high-risk PCa patients for whom neoadjuvant or adjuvant therapies should be explored, and (ii) the predictive/prognostic use of FDG-PET/CT in the early detection of metastatic recurrence where more effective combinatory approaches may be indicated.

#### 8.2.2. HP ^13^C Pyruvate MRSI

**Figure 7:**
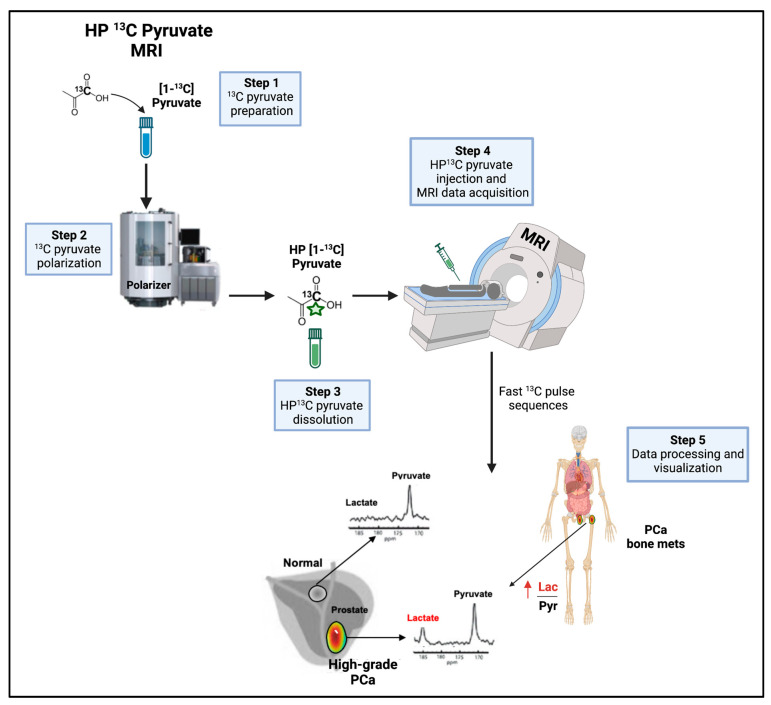
Non-invasive imaging of pyruvate-to-lactate conversion. Workflow of HP ^13^C pyruvate MRI in the clinical setting. HP ^13^C pyruvate MRI can provide useful information in the diagnostic workup and therapy decision making. HP = hyperpolarized, Lac = lactate, mets = metastases, MRI = magnetic resonance imaging, PCa = prostate cancer, Pyr = Pyruvate. Vertical red arrow means increase. This figure was created with BioRender.com with license rights.

Over the last two decades, the work of several groups worldwide has been groundbreaking in translating the application of HP [1-^13^C]pyruvate MRSI (Figure 7). The first-in-man imaging study was conducted in 2013 and successfully demonstrated the safety and feasibility of HP [1-^13^C]pyruvate MRSI as a tool to non-invasively detect metabolic alterations in PCa [132]. In 2020, a second pilot clinical study expanded the feasibility and imaging performance of HP [1-^13^C]pyruvate MRSI to bone and/or viscera PCa metastases in patients with metastastic CRPC [133]. Soon afterwards, an ex vivo MRSI approach was applied in patient-derived tissue slice cultures and confirmed the ability of HP ^1^[1-^13^C]pyruvate-to-lactate conversion in distinguishing high-Gleason versus low-Gleason PCa [40], setting the stage for MRSI as a non-invasive approach for patient stratification at diagnosis. These results have been further validated in two independent studies of PCa patients. In the first study, the authors reported increased ^13^C lactate accumulation and higher mRNA/protein levels of MCT-1 in high-Gleason primary PCa. These metabolic features were associated with PTEN mutations/deletions, highlighting lactate synthesis as a PTEN loss/mutation-specific metabolic vulnerability that can be exploited in precision medicine [134]. In the second study, the authors correlated ^13^C lactate labeling on MRI with the percentage of Gleason pattern four (an established marker of PCa aggressiveness) and were able to differentiate clinically significant PCa from indolent disease in a low/intermediate-risk population. The authors also uncovered a correlation between the [1-^13^C]lactate signal with both LDHA (epithelial) and MCT-4 (ratio of epithelium-to-stroma), highlighting the potential of HP ^13^C MRSI to stratify patients based on metabolic differences in the epithelial and stromal tumor compartments [135]. A Phase II trial has recently been initiated to evaluate the performance of HP [1-^13^C]pyruvate MRI in monitoring PCa patients under active surveillance (NCT03933670).

Consistent efforts have been devoted to evaluating HP [1-^13^C]pyruvate MRI performance in monitoring patient responses to treatment, including chemotherapy and ADT.

A significant k_PL_ decrease was reported in metastatic lesions from patients undergoing ADT or following treatment with taxane plus platinum, suggesting k_PL_ as an early metabolic biomarker of the response to ADT and chemotherapy [133,136].

More recently, the potential of HP ^13^C MRI to monitor the response to immunotherapy has also been investigated with promising results. Several osseus metastatic sites were imaged after treatment with the PD1 inhibitor pembrolizumab in a patient with metastatic CRPC characterized by an elevated mutation burden and microsatellite instability. In agreement with multiparametric MRI results, the serial k_PL_ in the osseous metastases indicated a complete response at the metabolic level. Since CT and bone scintigraphy are neither sensitive nor specific for monitoring the treatment response and are susceptible to the treatment flare phenomena, HP ^13^C MRI may represent a promising biomarker in bone metastases [137].

Aside from the use of HP ^13^C MRI/MRSI as a prognostic biomarker, the benefit of integrating HP ^13^C MRI in the diagnostic workup has been actively investigated. In this context, work has been conducted to establish the safety and technical feasibility of integrating a rapid, 1-min HP [1-^13^C]pyruvate MRI acquisition into the standard-of-care multiparametric MRI for guiding transrectal ultrasound fusion prostate biopsies [138]. The use of k_PL_ as a biomarker to guide fusion prostate biopsies was shown to be safe and technically feasible. The ability of HP ^13^C MRI to detect occult but clinically relevant tumor foci that would remain undetected by conventional prostate MRI has also emerged [138]. However, whether the addition of ^13^C MRI results in a significant improvement in diagnostic accuracy still needs to be established.

As technologies for generating/delivering HP agents are being improved and MR data acquisition sequences and hardware optimized, the establishment of HP MRSI capabilities in major medical centers will significantly advance the current management of PCa patients by improving biopsy collection, the identification of high-risk patients, treatment planning, and non-invasive real-time monitoring of the therapy response/resistance, as well as active surveillance. Several clinical trials are currently ongoing to evaluate these aspects (NCT04346225, NCT02844647, NCT02526368, NCT03933670, NCT03581500).

## 9. Targeting Lactate Metabolism: New Therapeutic Opportunities

The clinical translatability of targeting lactate metabolism as a therapeutic strategy in advanced cases is currently in the limelight. The genetic silencing or pharmacological inhibition of LDHA or MCT-1/MCT-4 with different compounds (i.e., FX11, oxamate, α-cyano-4-hydroxycinnamic acid) has shown promising results in metastatic CRPC cell lines and murine models [45,139,140]. The inhibition of LDHA and MCT-4 promotes the efficacy of both radiotherapy and chemotherapy (i.e., docetaxel) [141,142]. LDHA knockdown or pharmacological inhibition with the compound FX11 has been particularly effective in the treatment of ATM-deficient CRPC cells, suggesting that targeting lactate synthesis may be beneficial for patients with PARP-inhibitor-resistant metastatic CRPC [62]. Furthermore, the inhibition of lactate efflux using the PI3K inhibitor copanlisib improves TAM anti-cancer phagocytic activation and the efficacy of combined ADT/anti-PD1 antibody treatment, suggesting that targeting lactate metabolism may immunologically “turn on” PCa and improve the response to immune checkpoint inhibitors (ICIs) [73]. The modulation of lactate synthesis/efflux in combination with ICIs or adoptive T-cell therapy has indeed provided promising results in preclinical models of melanoma [143,144]. Diclofenac, a non-steroidal anti-inflammatory drug and MCT inhibitor, improved the response to anti-PD1 in B16 allografts [143]. The LDHA inhibitor GSK2837808A increased the susceptibility of tumor cells to autologous tumor-infiltrating lymphocytes (TIL)-mediated killing in B16 allografts [144]. The competitive LDHA inhibitor gossypol (AT-101) has shown promising results in combination with chemotherapy in metastatic CRPC patients, although the lowest recommended dose of gossypol and its precise toxicity profile need to be confirmed in further studies as reviewed in [145].

While these studies support the rationale for the use of inhibitors of lactate metabolism, they also highlight the need for the development of more potent and selective inhibitors. The first-in-class MCT-1 inhibitor AZD3965 was tested in patients with diffuse large B-cell lymphoma and Burkitt’s lymphoma (Phase I expansion study NCT01791595). The study showed that AZD3965 can be safely administered at 10 mg bid with confirmed target engagement [146].

Overall, the preclinical studies support the potential for exploiting lactate metabolism as an adjuvant therapy in combination with chemotherapy, anti-AR signaling agents, and immunotherapy in metastatic CRPC. The development of more potent and selective LDHA and MCT-4 inhibitors with pharmacokinetic and pharmacodynamic properties suitable for clinical translation will greatly benefit from the widespread availability of HP ^13^C MRI in hospitals, enabling staff to carefully select candidate patients and monitor therapy efficacy. Given the critical role of MCTs in red blood cells, activated T cells, and neurons, the systemic effects of MCTs inhibitors need to be carefully evaluated before clinical translation.

## 10. Future Prospective

Lactate has emerged as an important player in PCa progression. However, the investigation of lactate in PCa is still in its infancy. Thus far, the results have been mostly derived from in vitro experiments or in vivo evidence obtained by the combination of MRSI, steady-state metabolomics, ^13^C-labeling experiments, bulk gene expression data, and pharmacological or genetic modulation of LDHA and MCTs. While these experiments have been instrumental in uncovering lactate’s role in PCa progression, a clear understanding of lactate’s concentration, distribution, and function in human tissues is still missing. We anticipate that ^13^C-labeling experiments using patient-derived co-culture organoids models will further strengthen our current understanding of lactate shuttling between PCa and TME cells. We believe that the implementation of single-cell metabolomics [147,148] and the integration of spatial metabolomics/transcriptomics and in situ multiplexed TME characterization [149] in human prostate cancer specimens will be crucial to: (i) precisely measure lactate concentrations in the TME; (ii) understand how lactate mediates PCa cells–TME crosstalk; (iii) define the role of lactate in specific stromal populations (i.e., immune subpopulations); and (iv) better understand metabolic heterogeneity in PCa. Both systemic metabolism and the gut microbiota influence PCa progression. However, it is still unknown whether lactate plays a role in mediating their effects on PCa progression. A more comprehensive study of the microbiota metabolome in PCa patients subjected to different diets or metabolic conditions (i.e., obesity) is required.

## 11. Conclusions

Given the pleiotropic oncogenic effects of lactate on both PCa and cells in the TME, targeting lactate metabolism represents a strategy to both halt cancer cell dissemination and promote cancer immune surveillance. Reverting the immune suppressive phenotype using lactate metabolism inhibitors may enhance the response to ICIs, which has thus far been poor for PCa. Furthermore, the AR-dependent regulation of metabolic pathways offers opportunities for combined treatments of lactate metabolism inhibitors with AR-signaling inhibitors, including enzalutamide and abiraterone. The non-invasive measurement of [1-^13^C]pyruvate-to-lactate conversion predicts clinically relevant disease and holds potential as: (i) a non-invasive approach to stratify high-risk PCa patients, and (ii) a tool to monitor residual/significant disease in patients given treatment (including treatments with LDHA and MCT-4 inhibitors) or on active surveillance. 

## Abbreviations

ADT	Androgen deprivation therapy
AR	Androgen receptor
ATM	Ataxia-telangiectasia mutated
ATP	Adenosine triphosphate
BID	bis in die (twice a day)
BRCA 1	Breast cancer gene 1
BRCA 2	Breast cancer gene 2
CAFs	Cancer-associated fibroblasts
CaMKK2	Calcium/calmodulin-dependent kinase kinase 2
CRPC	Castration-resistant prostate cancer
CT	Computed tomography
DNA-PK	DNA-dependent protein kinase
ECM	Extracellular matrix
EMT	Epithelial-mesenchymal transition
ERK	Extracellular signal-regulated kinase
FAs	Fatty acids
FASN	Fatty acid synthase
^18^F-FDG	^18^F-fluorodeoxyglucose
FGFR1	Fibroblast growth factor receptor
GEMM	Genetically engineered mouse model
GLUT1	Glucose transporter 1
HCAR1	Hydroxycarboxylic acid receptor 1
HIF-1a	Hypoxia-inducible factor-1
HP	Hyperpolarized
HR-MAS	High resolution magic angle spinning
ICIs	Immune checkpoint inhibitors
LDHA	Lactate dehydrogenase A
MALDI-MSI	Matrix-assisted laser desorption/ionization-mass spectrometry imaging
MCTs	Monocarboxylate transporters
CAFs	Cancer-associated fibroblast
MRI	Magnetic resonance imaging
MRSI	Magnetic resonance spectroscopic imaging
MS	Mass spectrometry
NAD	Nicotinamide adenine dinucleotide
NADPH	Nicotinamide adenine dinucleotide phosphate
NDRG3	N-MYC downstream-regulated gene 3
NF-KB	Nuclear factor kappa-light-chain-enhancer of activated B cells
NK	Natural killer cells
NMR	Nuclear magnetic resonance
PARP	Poly(ADP-ribose)polymerase
PCa	Prostate cancer
PDX	Patient-derived xenografts
PET	Positron emission tomography
PHD2	HIF prolyl hydroxylase domain-2
PI3K	Phosphoinositide 3-kinase
PTEN	Phosphatase and tensin homolog
RB1	RB transcriptional corepressor 1
ROS	Reactive oxygen species
SCFAs	Short-chain fatty acids
SREBP	Sterol regulatory element-binding proteins
